# Neonatal care practices need to be further explored – Authors' reply

**DOI:** 10.1016/S2214-109X(21)00095-4

**Published:** 2021-05-18

**Authors:** Emma Sacks, Hedieh Mehrtash, Mamadou Dioulde Balde, Theresa Azonima Irinyenikan, Kwame Adu-Bonsaffoh, Thae Maung Maung, Özge Tunçalp

**Affiliations:** a Department of International Health, Johns Hopkins School of Public Health, Baltimore, MD, USA; b UNDP/UNFPA/UNICEF/WHO/World Bank Special Programme of Research, Development and Research Training in Human Reproduction, Department of Sexual and Reproductive Health and Research, World Health Organization, Geneva, Switzerland; c Cellule de Recherche en Sante de la Reproduction en Guinee, Conakry, Guinea; d Department of Obstetrics and Gynaecology, Faculty of Clinical Sciences, University of Medical Sciences, Ondo University of Medical Sciences Teaching Hospital, Akure, Nigeria; e Department of Obstetrics and Gynaecology, School of Medicine and Dentistry, University of Ghana, Accra, Ghana; f Department of Medical Research, Ministry of Health and Sports, Yangon, Myanmar

The field of experience of care for (including mistreatment of) neonates is relatively new. Our Article^1^ exploring neonatal care practices in a multicountry observational study provides a preliminary examination of occurrences and sets the stage for additional research that is crucial to this agenda. We thank Harsha Kumar H N for his keen interest in this topic and for raising important questions, especially about the type of facilities providing immediate postnatal care to neonates globally.

The facilities included in this study were public and provided care that was either free of charge or covered by national health insurance. Although the type of mistreatment can vary by type of facility, there is evidence that mistreatment can occur at both public and private facilities.^2^ Even in facilities that provide free health-care, families might be required to make informal payments.^3^ In some instances, families who demand accountability receive better care, but cases in which families are retaliated against or have feared retribution have also been documented.^4^ Therefore, it is important to apply standards for quality of care^5^ at every facility providing maternal and neonate care, and to have patients and providers work together to achieve this goal.

Mistreatment is probably due to a multitude of drivers, including the conditions and constraints of health-care systems.^6^ Health-care systems should be accountable to patients and providers; they should provide enabling and empowering environments to support health-care workers, including proper equipment, training, supervision, and supportive policies. For example, to support breastfeeding, facility structures and policies should allow the participation of partners or of other family members, restrict the promotion of breast-milk substitutes, and encourage rooming-in of neonates with their families.^7^ Investing in the physical and human resources of health-care systems is essential for both public and private facilities, and across levels of care.

Building on the WHO study on mistreatment of women,^8^ we were able to use the available multicountry data to explore factors associated with neonatal care practices with a robust modelling approach.^9^ Kumar highlights additional factors that will need to be explored in future studies, such as the influence of the cost of services, types of facilities, training of providers, women's employment status, counselling on breastfeeding, and other family health-care preferences. We are hopeful that our paper promotes future research on defining and measuring neonatal care practices and on identifying strategies and policies to improve care.

We declare no competing interests.
